# Targeting the Endoplasmic Reticulum Oxidoreductin-1 Alpha–Protein Disulfide Isomerase Redox Interface as a Therapeutic Strategy in Cancer

**DOI:** 10.3390/biomedicines14020263

**Published:** 2026-01-23

**Authors:** Kamilla Khojayeva, Aiym Zhussipbekkyzy, Dilbara Balkybayeva, Karakat Sabit, Lucia Rossetti Lopes, Kamila Sagatbekova, Assem Zhakupova, Mohamad Aljofan

**Affiliations:** 1 Department of Biomedical Sciences, School of Medicine, Nazarbayev University, Astana 010000, Kazakhstan; kamilla.khojayeva@nu.edu.kz (K.K.); aiym.zhussipbekkyzy@nu.edu.kz (A.Z.); dilbara.balkybayeva@nu.edu.kz (D.B.); karakat.sabit@nu.edu.kz (K.S.); kamila.sagatbekova@nu.edu.kz (K.S.); a.zhakupova@nu.edu.kz (A.Z.); 2 Department of Pharmacology, Institute of Biomedical Sciences, University of São Paulo, São Paulo 05508-900, Brazil; llopes@usp.br

**Keywords:** endoplasmic reticulum stress, ERO1α, PDI

## Abstract

The endoplasmic reticulum (ER) is critical in aiding cells in ensuring that proteins are folded and processed correctly, particularly during stressful situations. ER oxidoreductin-1 alpha (ERO1α) is an enzyme that is responsible for the formation of disulfide bonds during protein folding, along with protein disulfide isomerase (PDI). This redox pathway is often highly upregulated in cancer cells, allowing tumors to survive harsh conditions such as hypoxia and nutrient deprivation. This review discusses the role of the ERO1α–PDI system in cancer development through the regulation of oxidative stress, redox homeostasis, and tumor plasticity. It further shows the therapeutic potential of interrupting the ERO1α–PDI axis, which could lead to protein misfolding; enhanced generation of reactive oxygen species (ROS); and, eventually, cancer cell death.

## 1. Introduction

The endoplasmic reticulum (ER) is an organelle that plays a key role in protein folding and maturation of eukaryotic cells [1]. To be stable and active, many of the secreted and membrane proteins need disulfide bonds, which are covalent linkages between cysteine residues that stabilize tertiary and quaternary structures [2]. The formation of these bonds is a highly regulated process that is largely dependent on the oxidative environment of the ER and the coordinated action of specialized enzymes. Among these, ER oxidoreductin-1 alpha (ERO1α) and protein disulfide isomerases (PDIs) form a critical redox interface that ensures proper disulfide bond formation, thereby maintaining proteostasis within the ER [3].

Cancer cells are characterized by rapid proliferation, heightened metabolic demands, and an increased load of protein synthesis, all of which challenge the protein-folding capacity of the ER. To cope with this stress, cancer cells often upregulate components of the ER protein-folding machinery, including ERO1α and PDIs [4]. This adaptive response is a form of “non-oncogene addiction”, where tumor cells become dependent on non-mutated genes that are not classical oncogenes but are essential for maintaining malignant phenotypes under stressed conditions [5]. Elevated ERO1α expression has been observed in various cancers, including breast, lung, and cervical tumors, and correlates with poor prognosis [6]. Similarly, PDIs have been implicated in promoting tumor survival and angiogenesis [7].

Beyond protein folding, ERO1α activity generates reactive oxygen species (ROS)—primarily hydrogen peroxide (H_2_O_2_)—as by-products of disulfide bond formation [8]. Importantly, ROS can have dual roles in cancer: at moderate levels, H_2_O_2_ acts as a secondary signaling molecule, modulating pathways involved in proliferation, migration, and angiogenesis; at higher levels, ROS can overwhelm antioxidant defenses, causing oxidative damage and apoptosis [9]. Distinguishing these effects is crucial, as the direct impact of ERO1α on protein folding underpins proteostasis, whereas H_2_O_2_-mediated signaling represents a secondary consequence that can influence tumor behavior.

In this context, a detailed understanding of the molecular and structural basis of the ERO1α–PDI interaction, as well as its regulation under physiological and pathological conditions, is crucial for designing effective therapeutic strategies. This review explores the role of the ERO1α–PDI redox interface in cancer biology, summarizes recent advances in targeting this pathway, and discusses future perspectives for translating these insights into clinically viable treatments. By focusing on this unique vulnerability of cancer cells, novel therapies targeting ER oxidative protein folding hold the potential to complement existing anti-cancer treatment and improve patient outcomes.

## 2. Physiological Roles of PDI, ERO1α, and Their Redox Interface

### 2.1. Protein Disulfide Isomerases (PDIs)

The first PDI was identified in the early 1960s. This enzyme was found to be capable of oxidizing reduced bovine pancreatic ribonuclease and was first isolated from rat liver and pigeon and chicken pancreas [10]. PDIs constitute a large and functionally diverse family of thioredoxin superfamily proteins, comprising at least 21 members in mammals [11].

Protein disulfide isomerase A1 (PDIA1) was the first member of the PDI family to be identified. It comprises four domains—designated as a, b, b′, and a′—that are organized into a U-shaped conformation [12]. PDIA1 facilitates the formation, rearrangement, and reduction of disulfide bonds between cysteine residues in substrate proteins [13]. PDIA1 exhibits broad substrate specificity, likely due to an expansive substrate-binding surface within its b′ domain, while other PDI family members, such as ERp57 (PDIA3) and ERp72 (PDIA4), perform more specialized functions. The primary function of PDIA3 is to facilitate glycoprotein folding within the ER. In cooperation with lectin chaperones calnexin and calreticulin, PDIA3 controls the folding of newly synthesized cysteine-rich glycoproteins by catalyzing the formation and rearrangement of disulfide bonds between cysteine residues [14]. In contrast, PDIA4 has been proposed to compensate for PDIA3 deficiency in knockout cell models and is induced in ER stress [15]. The physiological roles and substrate selectivity of many PDIs remain poorly defined [11].

The primary role of PDIs in the endoplasmic reticulum is to catalyze oxidative protein folding by introducing, reshuffling, and reducing disulfide bonds in newly synthesized polypeptides. Through these thiol–disulfide exchange reactions, PDIs ensure that secretory and membrane proteins attain their correct three-dimensional structure and functional stability [16]. In addition to their enzymatic activity, PDIs act as molecular chaperones that prevent aggregation of unfolded or misfolded proteins, thereby safeguarding ER proteostasis [17]. PDIs dynamically cycle between oxidized and reduced states, enabling them to function as oxidases, reductases, or isomerases depending on cellular redox conditions and substrate requirements [18].

### 2.2. Endoplasmic Reticulum Oxidoreductin 1α (ERO1α)

ERO1α is a membrane-associated flavoprotein. ERO1α reoxidizes PDIs via thiol disulfide exchanges and is reoxidized by molecular oxygen via its FAD cofactor [19]. Humans also express ER oxidoreductin-1 beta (ERO1β), though ERO1α is more broadly expressed and responsive to stress signals. Patient cohort analyses suggest that ERO1α is more frequently upregulated than ERO1β in malignancies and, consequently, is associated with adverse patient outcomes [20].

Every catalytic cycle of ERO1α oxidation of PDI results in the production of H_2_O_2_ as a by-product [21]. This reaction helps in oxidative protein folding in the ER under normal conditions. Nevertheless, in cancer cells, the low concentrations of ROS produced in the process obtain other functions. Most tumors are oxygen-deprived, and the UPR is constantly expressed. In these circumstances, cells seem to depend more on ERO1α to support protein folding. The generated ROS help in the process of folding of pro-angiogenic proteins like vascular endothelial growth factor A (VEGF-A) and its receptor-like vascular endothelial growth factor receptor 2 (VEGFR2), which require correct disulfide bond formation to be released and active. Inhibition of ERO1α in experiments reduces VEGF-A release and disrupts endothelial tube differentiation, which demonstrates the relevance of ERO1α to tumor-related angiogenesis [22,23].

### 2.3. Functional Coupling of PDI and ERO1α in the Endoplasmic Reticulum

**Figure 1:**
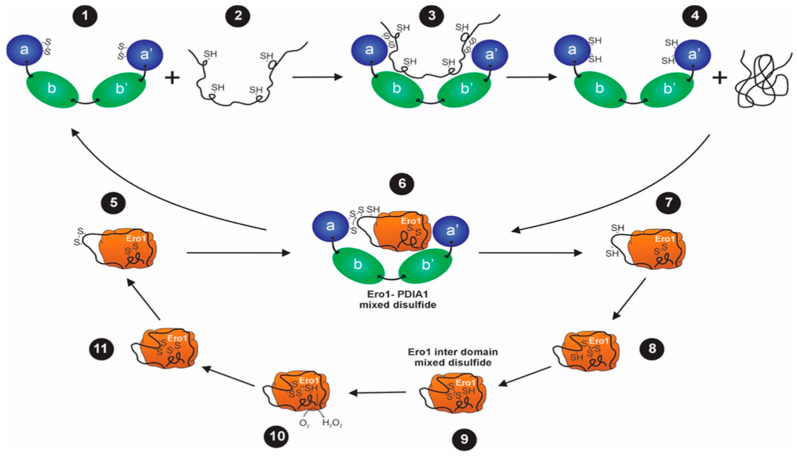
Schematic representation of reoxidation of protein disulfide isomerase A1 in the endoplasmic reticulum mediated by ERO1α. Nascent polypeptides are translocated into the lumen of the endoplasmic reticulum (ER), where they undergo oxidative folding. PDI catalyzes the formation of disulfide bonds within nascent proteins, converting them from the reduced (-SH) to the oxidized (S-S) state (1–4). During this reaction, PDI becomes reduced (4). Reduced PDI is subsequently reoxidized by endoplasmic reticulum oxidoreductin 1 alpha (ERO1α) through direct electron transfer (5,6). Electrons flow from the catalytic cysteines of PDI to the shuttle cysteines of ERO1α, then to its FAD cofactor (7–9). The reduced FAD transfers electrons to molecular oxygen (O_2_), generating hydrogen peroxide (H_2_O_2_) as a by-product (10). This cycle regenerates oxidized ERO1α and maintains a pool of oxidized PDI required for continuous disulfide bond formation in newly folded proteins (11). Adapted from Sevier and Kaiser, Mol. Biol. Cell 17, 2256–2266 (2006). https://doi.org/10.1091/mbc.e05-05-0417 [24].

The action of the ERO1α–PDI relay is based on a direct electron transfer chain: a reduced substrate passes electrons to PDIs; PDIs then transfer them to ERO1α, which then transfers it to molecular oxygen. In the catalysis process, PDIs are reduced and lose their activity unless they are re-oxidized. In mammalian cells, this reoxidation is primarily performed by ERO1α. It links the PDI cycle with molecular oxygen. Essentially, ERO1α has redox-active cysteines that receive electrons directly emitted by reduced PDIs and transfer them to its flavin adenine dinucleotide (FAD) cofactor, where oxygen is the ultimate acceptor. Consequently, this reaction chain generates hydrogen peroxide (H_2_O_2_) [8]. This relay maintains a store of oxidized PDIs that is accessible and enables continuous oxidative folding of nascent proteins (Figure 1).

**Figure 2:**
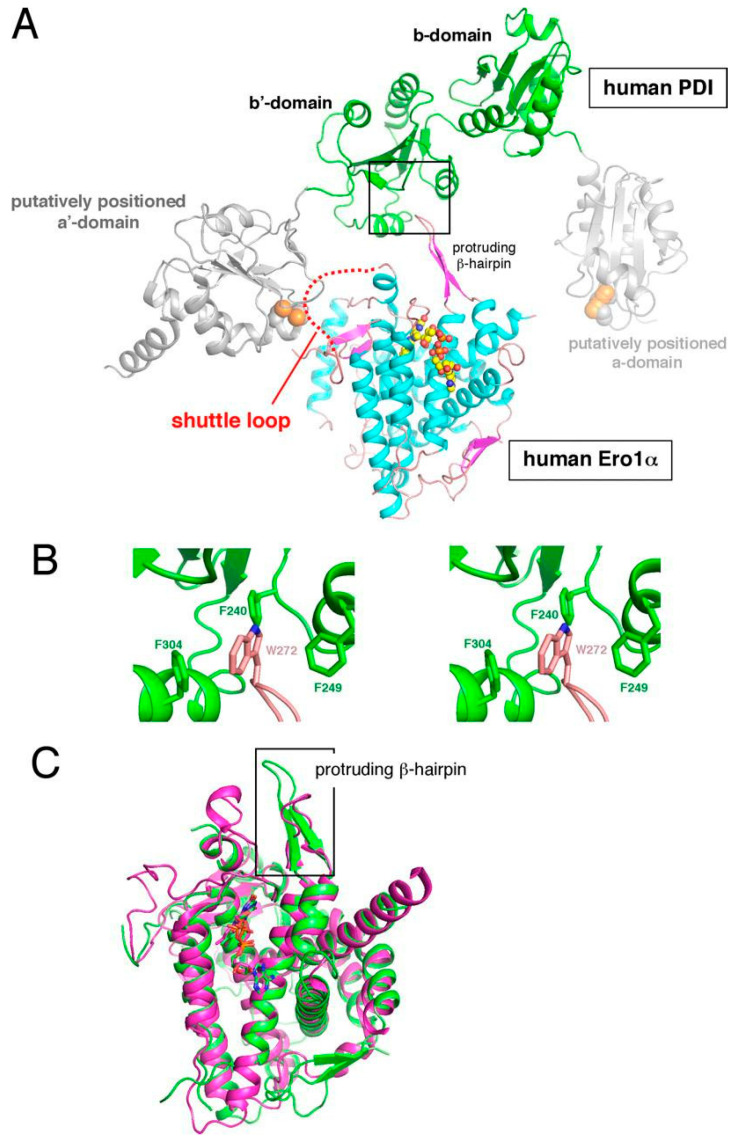
Human ERO1-PDI docking simulation. (**A**) Model generated with the SurFit server showing full-length human ERO1α docked with the b–b′ domains of human PDI. The FAD cofactor in ERO1α is shown in yellow–orange, and the boxed area highlights the contact region between the two proteins. (**B**) A closer view of the interface shows how ERO1α residue Trp272 interacts closely with PDI residues Phe240, Phe249, and Phe304 through non-covalent contacts. (**C**) Structural alignment of human (green, PDB 3AHQ) and yeast (magenta, PDB 2B5E) ERO1 shows an overall similar fold, but the β-hairpin in human ERO1α extends farther compared to the shorter one in yeast ERO1p. Figure reproduced from Masui et al., J. Biol. Chem. 286, 16261–16271 (2011) https://doi.org/10.1074/jbc.M111.231357, licensed under CC BY 4.0 [25].

Structural and biochemical mapping demonstrated that ERO1α has a β-hairpin loop that interacts with the b′ domain of PDI, which precisely positions its catalytic cysteines to undergo thiol–disulfide exchange [25] (Figure 2).

Considering this, the electron transfer in the ERO1α–PDI relay occurs via transient covalent states. In the context of catalysis, ERO1α and PDI create mixed disulfide reaction intermediates that serve as the hand-off points of the electronic transfer [21]. These covalent bridges are directional, and backflow of the electrons is prohibited. When the intermediate is resolved, PDI is reoxidized, and the relay proceeds [3]. Late formation or resolution of these intermediates leads to a buildup of reduced PDI, slowed oxidative folding, and ER stress [26]. This extra stress in tumor cells that are already on the edge of proteostasis can lead to survival or death.

The interaction also depends on the presence of specific residues of the ERO1α–PDI interface. In ERO1α Val101 was found to be an important hotspot in cervical cancer models in terms of identifying PDI. Val101 mutation undermines binding and decreases folding performance, as well as tumor growth and invasion, in mice [27]. This direct correlation of the behavior of a tumor with a single amino acid indicates why the interface is an attractive drug target. The complexity of the PDI family in terms of structural diversity also introduces a further therapeutic opportunity. The various paralogs of PDI have different substrate-binding groves and non-redundant functions in cancer. Recently, a review suggested that this diversity might be utilized to create interface-selective inhibitors that selectively deactivate ERO1α that prefers PDI partners in tumor cells and spares other PDIs that are required to sustain normal secretory activity [28].

This docking creates an active but highly regulated microenvironment that aligns the redox centers, reduces off-pathway reactions, and catalyzes the turnover quickly. In the therapeutic context, an intermediate between two extremes could be achieved by targeting the ERO1α–PDI docking interface. On the one hand, broad PDI inhibitors block the growth of tumors but can also affect other PDIs, causing toxicity in secretory tissues [29]. On the other hand, downstream antioxidant-only targeting is not sufficient to reduce oxidative folding throughput. An interface-specific blocker would act by inhibiting the physical interaction between ERO1α and PDI, which is the location of electron transfer. Those agents may block PDI reoxidation, decrease the folding flux, cause ER stress, and promote apoptosis in tumor cells—in particular, when used together with agents that enhance the oxidative burden or disrupt the proteostasis systems. In fact, activation of the UPR, as well as suppression of tumor growth, with broad-spectrum pancreatic cancer PDI inhibitors like E64FC26 has been demonstrated but with different kinds of death mechanisms depending on the cell line [30,31].

## 3. Dysregulation of PDI and ERO1α in Cancer

### 3.1. PDI Involvement in Cancer Progression

Many cancer types have been reported to overexpress members of the PDI family, with PDIA1 emerging as the most consistently upregulated isoform across solid tumors. It was shown that PDIA1 is one of the most frequently overexpressed proteins in the interstitial fluid, plasma, and blood because it is secreted into circulation by tumor cells [32]. This implies that PDIA1 may be used as a non-invasive biomarker for early cancer diagnosis. Besides that, Lee and Lee (2017) documented that PDI facilitates the activation of membrane-bound client proteins, including metalloproteases, selectins, and integrins, which further enhance tumor cell adhesion and invasion [33]. A summary of these findings is provided in Table 1.

PDI family members are consistently overexpressed across diverse cancer types, indicating that enhanced ER redox capacity is a common feature of malignant cells. This pattern is closely linked to hypoxia and chronic ER stress within the tumor microenvironment, which activate the unfolded protein response (UPR) and increase cellular dependence on PDI-mediated oxidative folding.

Functionally, elevated PDI expression supports key tumor-promoting phenotypes. In breast cancer, increased levels of ER folding enzymes correlate with stem-like characteristics and therapy resistance, in line with reports demonstrating that PDI overexpression enhances mammosphere formation and self-renewal capacity [35]. Similar adaptive roles are observed in lung cancer, with PDIA4 and PDIA6 selectively upregulated in cisplatin-resistant non-small cell lung cancer [37] and PDI expression positively correlated with ERO1α levels [38], suggesting coordinated regulation of ER redox machinery during drug resistance.

Beyond therapy adaptation, PDI also appears to contribute to tumor invasiveness. In glioma, elevated PDI expression at the invasive tumor margin is associated with enhanced migratory behavior, consistent with a role for ER stress adaptation in highly invasive cancer cells [34]. The functional relevance of PDI overexpression is supported by loss-of-function studies in ovarian cancer, where pharmacological inhibition or genetic silencing of PDI induces marked cytotoxicity, demonstrating that cancer cells rely on sustained PDI activity for survival [39].

Mechanistically, these effects are mediated through modulation of UPR signaling pathways. PDI functions within a chaperone network that includes GRP78, GRP94, and BiP—proteins frequently overexpressed in tumors to buffer ER stress and promote survival. Disruption of this network can activate PERK-dependent pro-apoptotic signaling via CHOP/GADD153, although PERK pathway outputs remain context-dependent, exerting either tumor-suppressive or tumor-promoting effects [40]. This context dependency is further illustrated in breast cancer, where PDIA1 silencing elicits distinct transcriptional programs in estrogen receptor-positive and triple-negative cells. RNA-seq analyses revealed that PDIA1 modulates carcinogenesis through divergent pathways depending on ERα status, influencing ROS-associated catabolic processes and G2/M cell cycle regulation in ERα-positive tumors while affecting NF-κB signaling, mitochondrial biogenesis, metabolic reprogramming, and immune-related pathways in ERα-negative breast cancer [36].

### 3.2. ERO1α in Cancer Biology

ERO1α is associated with multiple redox-sensitive signal transduction pathways in tumor and endothelial cells. Low concentrations of H_2_O_2_ may trigger kinases like phosphoinositide 3-kinase/protein kinase B (PI3K/Akt) and extracellular signal-regulated kinases 1 and 2 (ERK1/2) and stabilize HIF-1α, thereby stimulating gene expression that leads to vessel development and cell survival. These modifications are beneficial in increasing endothelial cell migration, tissue remodeling, and neovascularization, thereby promoting tumor growth [41]. To mitigate possible oxidative stress, cancer cells enhance their antioxidant capacity through the increased expression of antioxidants like peroxiredoxin-4 (PRDX4) and glutathione peroxidases 7 and 8 [42]. These enzymes remove excessive H_2_O_2_ but retain an oxidizing environment in the ER, which is favorable for protein folding. This interaction allows the ERO1α protein to be involved not only in the regulation of folding but also in redox homeostasis, angiogenesis, and tumor survival in a hypoxic environment.

Evidence from multiple cancer types illustrates how this balance supports tumor progression. In pancreatic cancer, ERO1α expression is rapidly induced by hypoxia and promotes tumor growth and progression in both in vitro and mouse models; genetic ablation of ERO1α reduces ROS levels, impairs proliferation, and restricts tumor growth, indicating a strong dependence of pancreatic tumors on ERO1α-mediated oxidative folding under low-oxygen conditions [43]. Similar mechanisms operate in breast cancer, where ERO1α enhances oxidative folding and VEGF-A production in MDA-MB-231 cells and xenograft models, driving angiogenesis and tumor growth, with high ERO1α expression correlating with poor prognosis in triple-negative breast cancer [44]. In cholangiocarcinoma, elevated ERO1α expression promotes proliferation, migration, and epithelial–mesenchymal transition (EMT) through Akt/mTOR signaling and is associated with adverse clinical outcomes [45].

ERO1α also contributes to tumor aggressiveness in colorectal cancer, where ERO1α-positive tumor epithelial cells drive invasion and EMT, and high expression levels predict poor patient outcomes [46]. Hypoxia-induced upregulation of ERO1α in colorectal cancer cells enhances integrin-β1 maturation and trafficking, thereby supporting cell motility and invasive behavior; conversely, ERO1α deficiency disrupts protein glycosylation and impairs migration, particularly under hypoxic conditions [47]. Large-scale analyses of tumor cell lines and tissues further support ERO1α as a robust endogenous marker of hypoxia, in some contexts outperforming established markers such as carbonic anhydrase IX (CA9) in delineating low-oxygen tumor regions [48].

Other studies implicate ERO1α in conferring resistance to ferroptosis. ERO1α aids in promoting the antioxidant defenses of tumors with active mechanistic target of rapamycin complex 1 (mTORC1) signaling by promoting cystine uptake through SLC7A11. To this end, the inhibition of ERO1α makes these tumors susceptible to ferroptosis, indicating that oxidative folding is involved not only in facilitating protein maturation but also redox-dependent survival [49].

ERO1α-driven redox signaling also contributes to immune evasion and therapy resistance. In hepatocellular carcinoma, ERO1α activates the S1PR1/STAT3/VEGF-A axis to promote migration and angiogenesis, with elevated expression correlating with reduced survival [50]. In lung adenocarcinoma, overexpression of ERO1α is associated with a hypoxia-induced, immunity-suppressive tumor microenvironment and predicts resistance to immunotherapy [51], whereas genetic deletion of ERO1α enhances anti-tumor immunity, induces immunogenic cell death, and improves responses to immunotherapy in experimental models [52]. Consistent with these findings, bioinformatic analyses across lung cancer subtypes identify ERO1α as a negative prognostic factor with potential use for therapy stratification [53]. In pancreatic ductal adenocarcinoma, combined inhibition of ERO1α and IDO1 enhances dendritic cell infiltration and limits tumor growth, linking ERO1α activity to immune regulation within the tumor microenvironment [54].

Comparable patterns are observed in prostate and cervical cancers. In prostate cancer, elevated ERO1α expression correlates with higher Gleason scores, and gene silencing reduces proliferation, invasion, and integrin-β1 expression, highlighting its role in matrix interaction and tumor progression [55]. In cervical cancer, ERO1α promotes tumor growth, migration, and EMT through H_2_O_2_-mediated signaling; notably, the Val101 residue is essential for ERO1α–PDI interaction and oxidase activity, and its mutation suppresses oxidative folding and tumor development in vivo, directly linking the ERO1α–PDI interface to malignant behavior [27].

Collectively, these findings, along with their integrated summary in Table 2, consistently demonstrate that ERO1α is a key catalyst in oxidative protein folding but also a more general regulator of tumor adaptation, redox control, and clinical outcomes.

## 4. Small Molecule and Peptide Inhibitors of ERO1α and PDI

ERO1α continuously reoxidizes PDI to maintain disulfide bond formation, a process that directly supports the oxidative folding capacity of the endoplasmic reticulum. Cancer cells—particularly those with high secretory activity, hypoxic tumor microenvironments, or activated UPR pathways—rely heavily on this oxidative machinery [20]. This dependence creates a potential therapeutic window: inhibiting ERO1α or PDI preferentially disrupts tumor cell homeostasis, while most normal cells can tolerate the resulting ER stress without significant cytotoxicity.

### 4.1. PDI Inhibitors

Compared to ERO1α inhibitors, PDI-targeting pharmacology is more developed, largely because PDI proteins participate in multiple cancer-associated processes [7], including integrin maturation [56] and MHC-I peptide loading [57]. However, the PDI family includes more than 20 isoforms [58], and most inhibitors lack isoform selectivity [59].

The PACMA (propynoic acid carbamoyl methyl amide) family, with PACMA31 as the most well-characterized member, represents a class of electrophilic small molecules that covalently target the active-site cysteines of PDIs—primarily PDIA1 [60]. In vitro, PACMA31 efficiently inhibits PDI activity, induces accumulation of misfolded proteins in the ER, triggers the unfolded protein response (UPR), and promotes apoptotic cell death. In vivo, PACMA31 exhibits significant anti-tumor efficacy in ovarian cancer xenograft models, reducing tumor growth without causing overt systemic toxicity [39].

16F16 is an irreversible small molecule inhibitor of PDI that covalently modifies free cysteine thiols to block its reductase activity, as first described by Hoffstrom et al. [61]. In MDA-MB-231 and HCC1937, 16F16 reduced cell adhesion, spreading, and the formation of pro-migratory F-actin structures. Treatment also impaired cell migration in scratch-wound assays, with effects more pronounced in basal-like cell lines compared with PACMA-31, which preferentially inhibits PDIA1 [62].

RB-11-ca was identified as the first characterized a-domain-selective inhibitor of PDIA1, binding specifically to Cys53, the N-terminal catalytic cysteine of the a domain. In vitro fluorescence-based activity assays showed that RB-11-ca inhibits PDI activity comparably to commercially available inhibitor 16F16 and decreases the proliferation of HeLa cells [63].

Cole et al. identified KSC-34 as a highly potent and exceptionally selective PDIA1 inhibitor derived from lead compound RB-11-ca. KSC-34 covalently targets Cys53 in the a domain and incorporates an alkyne handle that enables rapid assessment of potency and selectivity in living cells. It is ~38-fold more potent than 16F16 and shows strong selectivity for PDIA1 within the cellular proteome. Importantly, in MCF-7 cells, KSC-34 caused only minimal and transient activation of the IRE1α arm of the UPR, indicating limited off-target stress responses [13].

**Figure 3:**
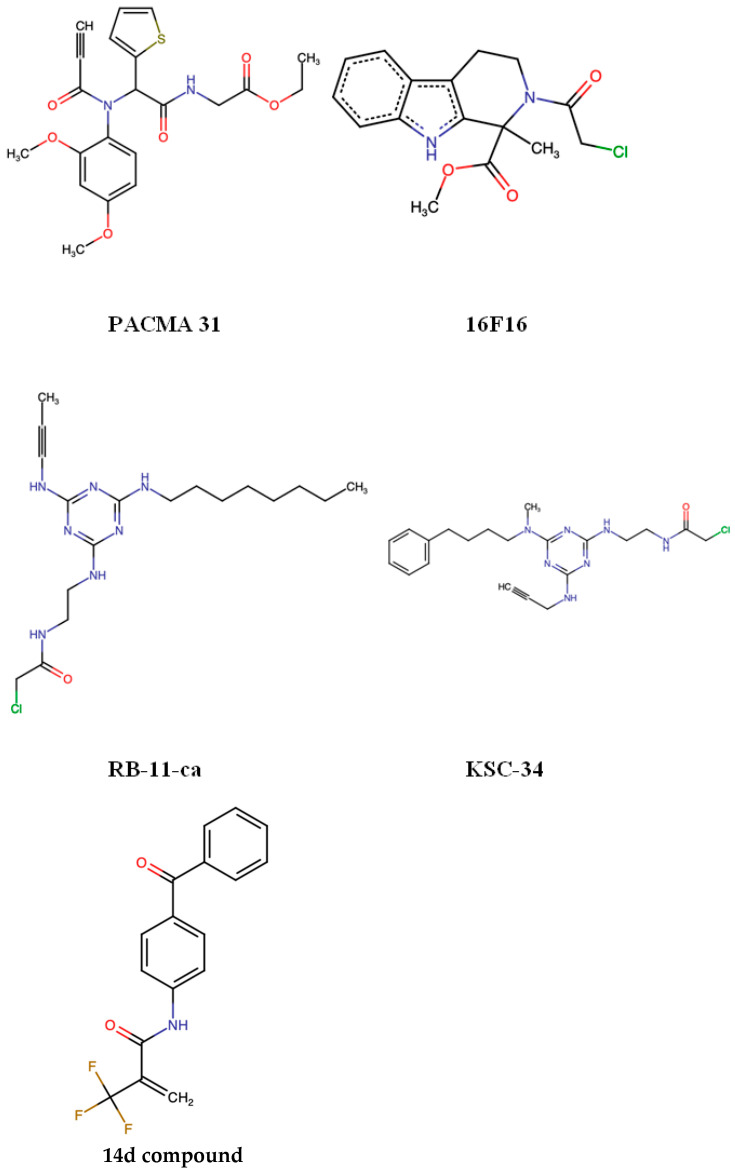
Chemical structure of PDI inhibitors.

Tseng and colleagues recently identified PS1, a selective PDIA4 inhibitor that improved multiple diabetic parameters in db/db mice by reducing ROS and protecting β-cell function [64]. Building on related PDI-targeting strategies, Chu et al. developed compound 14d, a highly potent PDIA1 inhibitor derived from warhead optimization, which effectively suppressed platelet aggregation and thrombosis without cytotoxicity [65]. Both inhibitors demonstrated strong activity in non-cancer disease models, and neither has yet been tested in cancer, leaving open the possibility of future oncologic applications. The chemical structures of the PDI inhibitors are represented in Figure 3.

An overview of PDI-targeting compounds, including their isoform selectivity, experimental validation, and clinical status, is summarized in Table 3.

### 4.2. ERO1α Inhibitors

EN460 remains the most widely characterized small molecule ERO1α inhibitor, originally identified together with QM295 in the seminal biochemical screening study by Blais et al., which first demonstrated that ERO1α could be pharmacologically targeted [67]. Originally identified through screening of quinazolinone derivatives, EN460 acts as a covalent or quasi-covalent modifier of the FAD proximal active site of ERO1α, reducing its catalytic turnover rate [67]. However, EN460 lacks specificity: it inhibits other flavin-dependent oxidoreductases, including MAO-A/B, raising concerns about off-target toxicity [68]. Despite these limitations, EN460 remains the primary pharmacological probe to study ERO1α biology. QM295, discovered alongside EN460 in the same study, is a reversible thiol-reactive inhibitor with good in vitro potency, yet it similarly shows limited cellular efficacy and some nonspecific cytotoxicity [67].

Varone et al. clarified that although EN460 inhibits ERO1α in the low micromolar range, its Michael acceptor electrophile allows for reaction with cellular thiols like glutathione, raising concerns about selectivity and off-target effects. To address this, the authors performed structure–activity optimization and identified improved EN460 analogs I2 and I3, which bind ERO1α directly, more effectively trap it in a reduced inactive state, and suppress VEGF-A secretion. Notably, I2 showed superior activity in TNBC models, reducing VEGF-A and PD-L1 in tumors without detectable toxicity [69].

**Figure 4:**
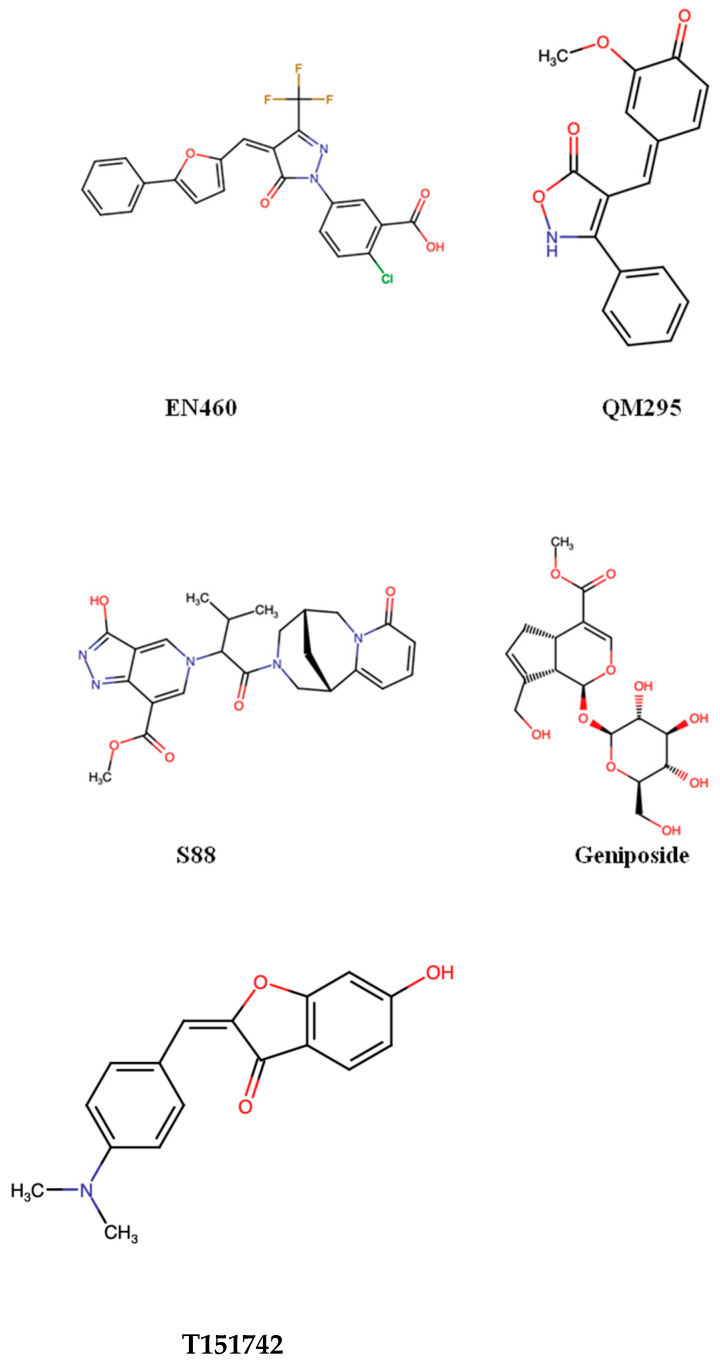
Chemical structure of ERO1α inhibitors.

A recent large-scale natural-product screening [70] identified two new ERO1α inhibitors—S88 and geniposide—that improved neuromuscular function in ALS and congenital muscle disorder models. Although they have not yet been tested in cancer, they highlight the possibility of discovering therapeutically relevant ERO1α inhibitors for oncology. Another study by Johnson et al. [71] revealed that T151742, the most potent aurone analog identified to date, selectively inhibits ERO1α in recombinant and cell-based assays and impairs clonogenic growth in vitro. Although it has not yet been evaluated in vivo, its specificity suggests strong potential for future cancer studies. The chemical structures of ERO1α inhibitors are represented in Figure 4.

The pharmacological landscape of ERO1α inhibition, encompassing biochemical, cellular, and in vivo validation, is outlined in Table 4.

**Figure 5:**
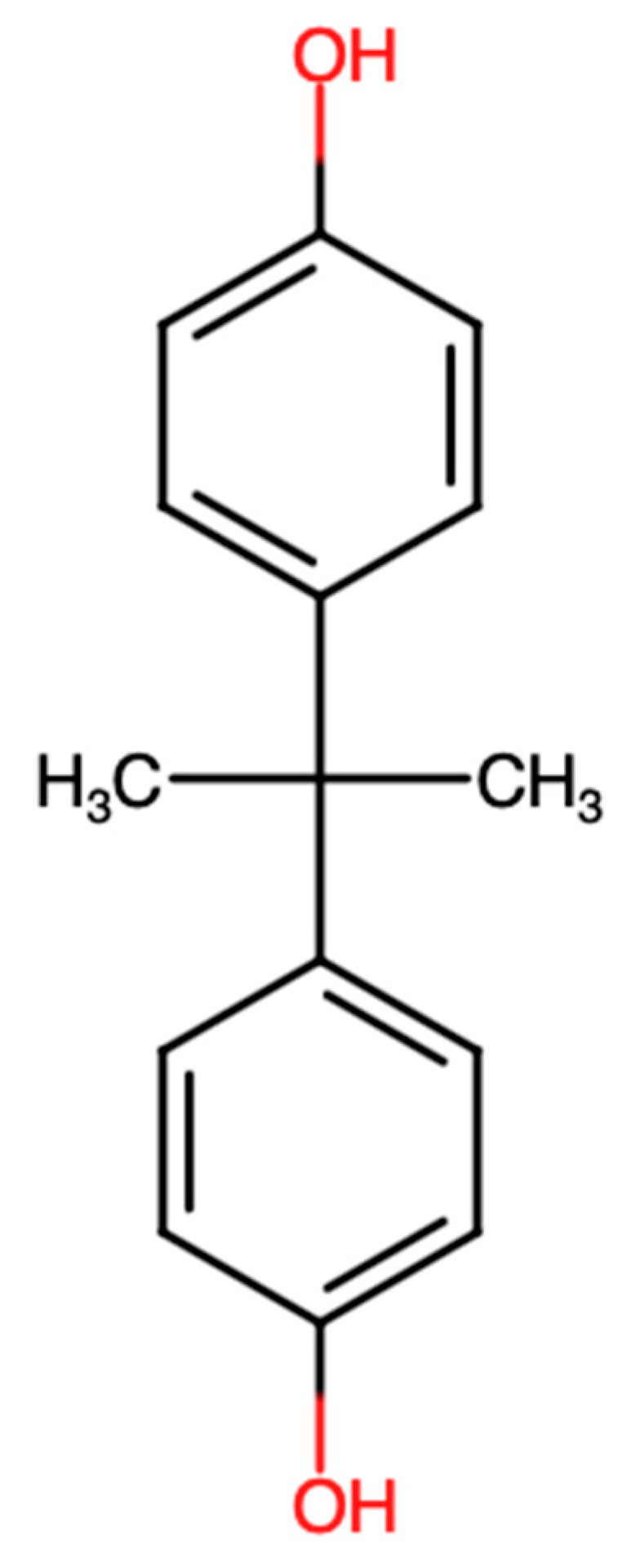
Chemical structure of bisphenol A.

Unlike inhibitors that target ERO1α or PDI alone, there is limited literature suggesting that small molecules could interfere with direct functional interaction between ERO1α and PDI. An example is bisphenol A, which was demonstrated to disrupt ERO1α–PDI-dependent disulfide transfer by disrupting their redox interaction, resulting in lower oxidative folding activity in vitro. Even though bisphenol A is not selective and cannot be used as a therapeutic compound, its activity offers valuable proof of concept that the ERO1α–PDI interface is chemically addressable [72]. The chemical structure of bisphenol A is represented in Figure 5.

Taken together, these studies argue in favor of the idea that interfering with the ERO1α–PDI redox interface, as opposed to blocking either enzyme on its own, is a unique mechanistic approach. Nevertheless, there is a scarcity of verified interface-directed inhibitors, which should be replaced by additional structure-driven discovery programs.

## 5. Translational and Clinical Implications

In many tumor types, ERO1α and PDI have emerged as prognostic biomarkers based on patient-derived correlative data. Clinical cohort analyses show that elevated ERO1α expression is associated with poor outcomes in several cancers. For example, Zhang et al. (2025) showed that high expression of ERO1α in lung cancer is associated with lower survival and activation of pathways such as epithelial–mesenchymal transition, PI3K/AKT/mTOR, and IL-6/JAK/STAT3, which directly contribute to tumor growth and aggressiveness [73]. Furthermore, regulation of ERO1α by the AC087588.2/miR-30a-5p pathway is linked to an increased immune response and reduced patient survival, so this indicator is also helpful in prediction. The same conclusions were made about pancreatic cancer: ERO1α is a part of the signature of ER stress genes, and its increased expression correlates with reduced survival rates [74]. In addition, PDIA3, an ER redox regulator, is also more frequently expressed in different cancer types and is a marker of poor clinical outcomes and limited response to immunotherapy [75].

In contrast, functional and causal evidence for ERO1α–PDI involvement in therapy response is derived from preclinical studies. Experimental models demonstrate that ERO1α-mediated oxidative protein folding stabilizes drug-resistance transporters such as ATP-binding cassette subfamily G member 2 (ABCG2) and multidrug resistance-associated protein 1 (MRP1), whereas disruption of disulfide bond formation sensitizes tumor cells to cytotoxic agents [76]. In addition, activity of the ERO1α–PDI system generates hydrogen peroxide, thereby modulating intracellular oxidative stress levels and contributing to redox-dependent drug resistance pathways [77]. More broadly, ER stress sensors, including GRP78, PERK, IRE1, ATF6, and PDI, enable tumor cells to adapt to hypoxia and therapeutic pressure, highlighting these pathways as potential targets for combination strategies [78].

Preclinical intervention studies further support a causal role for the ERO1α–PDI axis in tumor progression and immune modulation. Benham et al. provided evidence that inhibition of ERO1α–PDI delays protein folding in tumor cells and inhibits VEGF-A angiogenesis and immune signaling pathways [79]. Loss of ERO1α causes tumor cells to become more apparent to the immune system. Consequently, infiltration of CD8+ T cells and the release of DAMPs increase, which enhances the response to immune checkpoint inhibitors and can even stimulate immune memory development [52]. ERO1α blockers, in combination with ER stress-inducing drugs such as proteasome blockers or PERK and ATF6 inhibitors, additionally suppresses the ERK1/2-mTOR pathway [80]. There is also a link between ERO1α and angiogenesis, as the NFIB–ERO1α axis stabilizes HIF-1α, and knockdown of peroxisome proliferator-activated receptor delta (PPARδ) results in increased VEGF-A [81,82].

## 6. Challenges and Future Perspectives

The challenges of targeting ERO1α in the treatment of cancer involve both scientific and practical aspects that need to be considered to make this treatment strategy clinically viable. Redundancy in the ER oxidative folding network is one of the initial problems in targeting ERO1α. ERO1β has the ability to replace a few functions, and other enzymes, including peroxiredoxin IV and glutathione peroxidases 7 and 8, are also involved in disulfide bond formation. The PDI family is also large, and its paralogs overlap in their activities. Consequently, inhibition of ERO1α might not be sufficient to inhibit oxidative folding because cancer cells might also evolve and use other oxidases, or they may switch to other PDIs [20]. Combination strategies are under consideration to resolve this. Some such solutions involve co-inhibiting an ERO1α–PDI interface with an inhibitor that is selective to a tumor-relevant PDI isoform. Another approach is to pair ERO1α inhibitors with ferroptotic inducers, which can take advantage of compromised antioxidant responses in tumors with active mTORC1 signaling [49].

Safety is another concern. Oxidative folding is necessary to generate key proteins important to normal secretory cells like insulin in pancreatic β cells, albumin in hepatocytes, and antibodies in plasma cells. Blocking ERO1α or PDIs excessively may overwhelm cells under ER stress and disrupt the functioning of organs. Reviews of PDI-targeted agents underline this risk and indicate the necessity of selective or tumor-targeted strategies [29]. Technologies that enhance delivery, like nanoparticles, antibody drug conjugates, and hypoxia-activated prodrugs, provide alternatives to localize drug activity to the tumor microenvironment in order to reduce systemic toxicity. On a more general level, ER stress research in cancer warns that induction of global stress in all tissues is a double-edged sword; in many cases, it is detrimental, inducing ER stress in all tissues [83], although it may be safer to selectively impair the ERO1α–PDI interface.

Structural knowledge is another weakness. Although the mapping of major surfaces has improved, high-resolution structures of the human ERO1α–PDI complex in active states are yet to be defined. This is a loophole that slows down the design of interface-directed drugs. The discovery that Val101 is essential in binding to PDI, which is an example of a mutational study, demonstrates that single residues can serve as functional hotspots [27]. As such, structural templates capturing docking geometries and mixed-disulfide intermediates would be useful to medicinal chemistry, which might need to be prioritized as the first step in the development of a roadmap to decipher/validate the “ERO1α–PDI interface” as a potential treatment strategy. Thus, transient states might be stabilized and visualized using techniques such as cryo-EM, NMR, and cross-linking mass spectrometry, along with biochemical mapping of important contact points. Such information could be used for in silico screening, fragment-based discovery, and the rational design of peptidomimetics [25].

Another challenge—and opportunity—is the selection of appropriate cancer settings and therapeutic partners. To determine cases in which the dependence on ERO1α is greatest, preclinical work should focus on comparing tumor cells with corresponding normal cells under hypoxia and high secretory loads. Therapeutic windows can be defined with the help of CRISPR-based knockouts or inducible knockdowns [47]. The newly designed chemical probes that will inhibit the docking of ERO1α to PDI should be tested by binding assays and in situ methods, thermal-shift or SPR readouts, and cell-based markers of BiP and CHOP induction, as well as tests of secretion efficiency and ROS accumulation [30,84]. In this context, combination studies appear to be particularly promising. As an example, the combination of ERO1α inhibitors and proteasome inhibitors or immune checkpoint blockade may enhance stress or immune recognition. Outside of redox balance, ERO1α has been found to mediate immune evasion, such as that of PD-L1 and myeloid cell repression, and there is a possibility that the disruption of this relay might enhance the success of immunotherapy against some cancers [85].

Lastly, clinical translation will be dependent on patient selection. The most sensitive tumors are likely to be those with high ERO1α and hypoxia levels or those with high levels of secretory demands, for example, pancreatic ductal adenocarcinoma, hepatocellular carcinoma, cervical cancer, and subsets of colorectal and lung cancers. Diagnostic tools such as ERO1α immunohistochemistry, used alongside hypoxia-related gene signatures or imaging, could help identify patients for inclusion in early-phase clinical trials. Interface-directed drugs may be used alongside oxidative burden-increasing or proteostasis-inhibiting therapies in clinical practice, and close attention to functions of the pancreas, liver, and immune compartments would assist in managing the toxicity risk [29].

## 7. Conclusions

The ERO1α–PDI system is a central regulator of oxidative protein folding in the endoplasmic reticulum and is frequently hyperactivated in cancer to sustain proliferation, survival under stress, angiogenesis, and resistance to therapy. When the interaction between ERO1α and PDI is altered, tumor cells can become more susceptible to chemotherapy and immunotherapy and face additional stress because of protein folding issues. Importantly, disrupting the interaction between ERO1α and PDI appears to be a more promising strategy than broadly inhibiting ER folding enzymes, as it selectively impairs proteostasis and increases stress in tumor cells. Although the structural organization of the ERO1α–PDI complex is not yet fully resolved and systemic inhibition may pose toxicity risks, advances in structure-guided drug design and tumor-selective delivery approaches offer feasible paths forward. A new approach would be especially promising for treatment of patients with hypoxic tumors, high secretory activity, or increased ERO1α-level tumors. Overall, targeting the ERO1α–PDI system appears to be a viable approach to enhance anti-cancer therapy.

## Figures and Tables

**Table 1:** Open-access evidence for PDI’s role in cancer.

Cancer Type	Main Findings	References (Open Access)
Glioma	PDI is highly expressed in invasive and migrating glioma cells in xenograft models and human glioblastoma tissues.	[34]
Breast cancer	PDIA1, PDIA3, PDIA4, and PDIA6 are upregulated, with PDIA1 detected in tumor tissue, interstitial fluid, plasma, and blood; Overexpression of PDI is associated with mammosphere formation and therapy-resistant cell populations; PDIA1 silencing in ERα-positive and ERα-negative cells alters gene expression related to ROS regulation, cell cycle control, immune signaling, metabolism, and calcium homeostasis.	[29,35,36]
Lung cancer	PDIA4 and PDIA6 are overexpressed in cisplatin-resistant non-small cell lung cancer cells, and PDI expression correlates positively with ERO1α levels.	[37,38]
Ovarian cancer	Pharmacological inhibition or siRNA-mediated silencing of PDI induces cytotoxicity in human ovarian cancer cell lines.	[39]

**Table 2:** Open-access evidence for ERO1α’s role in cancer.

Cancer Type	Main Findings	References (Open Access)
Pancreatic cancer	ERO1α expression was induced under hypoxic conditions and was associated with increased tumor growth and progression in pancreatic cancer cell and mouse models. Combined inhibition of ERO1α and IDO1 increased dendritic cell infiltration and reduced tumor growth.	[43,54]
Breast cancer	ERO1α promoted tumor growth and angiogenesis through oxidative protein folding and increased VEGF-A expression in MDA-MB-231 cells and mouse models. Elevated ERO1α expression was correlated with poor prognosis in triple-negative breast cancer.	[44]
Liver cancer	ERO1α activated the S1PR1/STAT3/VEGF-A signaling pathway, enhancing tumor cell migration and angiogenesis. Increased ERO1α expression was associated with reduced patient survival.	[50]
Lung cancer	ERO1α contributed to an immunosuppressive tumor microenvironment and reduced sensitivity to PD-1 blockade in lung cancer models. Genetic ablation of ERO1α enhanced T-cell-mediated anti-tumor immunity and induced immunogenic cell death. Elevated ERO1α expression was identified as a negative prognostic factor across lung cancer subtypes.	[52,53]
Prostate cancer	ERO1α expression positively correlated with Gleason score and was further increased in docetaxel-resistant prostate cancer cells. Genetic suppression of ERO1α reduced proliferation, migration, and invasion, with stronger effects observed in highly metastatic cells.	[55]
Cervical cancer	ERO1α promoted tumor growth, migration, and epithelial–mesenchymal transition through H_2_O_2_-dependent signaling. Mutation of Val101 disrupted ERO1α–PDI interaction, reduced oxidase activity, and suppressed oxidative folding and tumor progression.	[27]
Cholangiocarcinoma	High ERO1α expression enhanced proliferation, migration, and epithelial–mesenchymal transition via Akt/mTOR signaling and was associated with poor prognosis.	[45]
Colorectal cancer	ERO1α-positive cells exhibited increased invasive capacity and epithelial–mesenchymal transition, and high ERO1α expression was associated with unfavorable clinical outcome.	[46]

**Table 3:** PDI inhibitors and their experimental and clinical evaluation.

Compound	Primary PDI Isoform/Selectivity	Evidence from Isolated Enzyme Assays	Evidence from Cell-Based Assays	Evidence from Animal Studies	Clinical Evaluation Status	Reference
PACMA31	Predominantly PDIA1/P4HB; irreversible covalent active-site modifier	Forms covalent adducts with active-site cysteines → sustained inhibition of PDI activity	Reported to suppress ovarian cancer cell survival/proliferation and trigger ER stress/UPR-associated effects in tumor context	Xenograft efficacy (ovarian cancer mouse xenograft; tumor targeting + tumor growth suppression)	No clinical studies reported	[39]
16F16	Often used as PDIA1/PDI inhibitor; described as irreversible/thiol-reactive in the literature	Reported to inhibit PDI via interaction with catalytic thiols (commonly treated as irreversible in mechanistic discussions)	Widely used in cells to perturb PDI-dependent folding/stress pathways (many applications; mechanism varies by context)	In vivo use exists mainly in thrombosis/inflammation literature (blocking PDI-dependent TF activation in disease models); not established as an anti-cancer compound	No clinical studies reported	[66]
RB-11-ca	PDIA1 (a-domain) active-site covalent probe; binds Cys53 (N-terminal cysteine of the a-domain CXXC motif)	Target ID + site mapping supports selective covalent labeling of PDIA1 C53	Used as a cell-active covalent probe (cellular profiling/labeling); not primarily an in vivo efficacy molecule	No animal efficacy reported in the primary characterization paper	No clinical studies reported	[13]
KSC-34	PDIA1 a-site selective covalent inhibitor; reported ~30-fold selectivity for a vs. a′ site	Time-dependent inhibition reported with kinact/KI and a-site preference	Cellular characterization: minimal sustained UPR/ER stress relative to broader PDI inhibition; used as a selective PDIA1 tool	No in vivo evaluation reported in the primary characterization	No clinical studies reported	[13]
PS1	PDIA4/ERp72-targeting small molecule inhibitor used as a PDIA4-directed chemical probe	Mechanistic/pharmacologic characterization (PDIA4-focused)	Improves β-cell survival/ER stress phenotypes in cellular models	In db/db mice, PS1 (alone ± metformin) improves diabetic endpoints (GTT, HbA1c, incidence, and β-cell function markers)	No clinical studies reported	[64]
14d	Reported as a reversible PDI inhibitor	Reported IC_50_ ≈ 0.48 μM for PDI inhibition	Reduces platelet activation/aggregation with low cytotoxicity in the reported evaluation	Reduced thrombus formation in animal thrombosis models	No clinical studies reported	[65]

**Table 4:** ERO1α inhibitors and their experimental and clinical evaluation.

Compound	Primary Target/Selectivity	Isolated Enzyme Evidence	Cell-Based Evidence	Animal Evidence	Clinical Evaluation Status	References
EN460	ERO1α inhibitor; thiol-reactive tool compound	Reported IC_50_ of ~1.9 μM in original biochemical work	In multiple myeloma cells, EN460 inhibits proliferation/induces apoptosis (Hayes 2019 [68], PMC6554731) (PMC)	EN460 used in vivo and linked to VEGF/PD-L1/tumor effects	No clinical studies reported	[72,73,74]
QM295	Reported ERO1 inhibitor hit (often cited alongside EN460);	IC_50_ of ~1.9 μM in kinetic assay (vendor page; not primary literature)	Reported as HTS hit used for UPR/ERO1 pathway probing in literature reviews	Not reported	No clinical studies reported	[19]
I2/I3	Covalent ERO1α inhibitors	IC_50_: I2, ~8.1 μM; I3, ~3.5 μM	Cellular pathway effects (VEGF-A/PD-L1 axis)	In vivo testing consistent with anti-tumor pathway modulation	No clinical studies reported	[69]
T151742	Aurone/sulfuretin-derived ERO1α inhibitor; selectivity profiling included	Recombinant assay IC_50_ = 8.27 ± 2.33 μM; compared vs. EN-460	Cell viability IC_50_ of ~16 μM in MTT assay context	Not reported	No clinical studies reported	[71]
S88/Geniposide	Reported as ERO1α inhibitors	Inhibit ERO1α in vitro and reduce ER stress markers in neurons	Reduced tunicamycin-induced ER stress markers in human neurons	Improved Drosophila phenotypes/aging measures	No clinical studies reported	[70]

## Data Availability

No new data were created or analyzed in this study. Data sharing is not applicable to this article.

## Abbreviations

The following abbreviations are used in this manuscript:

ABCG2	ATP-binding cassette subfamily G member 2
Akt	Protein kinase B
ALS	Amyotrophic lateral sclerosis
ATF6	Activating transcription factor 6
BiP	Binding immunoglobulin protein
BPA	Bisphenol A
CA9	Carbonic anhydrase IX
CHOP	C/EBP homologous protein (GADD153)
CNX	Calnexin
CRT	Calreticulin
DAMPs	Damage-associated molecular patterns
EMT	Epithelial–mesenchymal transition
EN460	Small molecule ERO1α inhibitor
ER	Endoplasmic reticulum
ERK1/2	Extracellular signal-regulated kinases 1 and 2
ERO1α	Endoplasmic reticulum oxidoreductin 1 alpha
ERO1β	Endoplasmic reticulum oxidoreductin 1 beta
ERp57	Endoplasmic reticulum protein 57 (PDIA3)
ERp72	Endoplasmic reticulum protein 72 (PDIA4)
FAD	Flavin adenine dinucleotide
GADD153	Growth arrest and DNA damage–inducible protein 153
GRP78	Glucose-regulated protein 78
GRP94	Glucose-regulated protein 94
GSH	Reduced glutathione
GSSG	Oxidized glutathione
H_2_O_2_	Hydrogen peroxide
HCC	Hepatocellular carcinoma
HIF-1α	Hypoxia-inducible factor 1 alpha
HTS	High-throughput screening
IDO1	Indoleamine 2,3-dioxygenase 1
IRE1	Inositol-requiring enzyme 1
MAO-A/B	Monoamine oxidase A/B
MCF-7	Human breast cancer cell line
MDA-MB-231	Triple-negative breast cancer cell line
MHC-I	Major histocompatibility complex class I
MPR1	Multidrug resistance-associated protein 1
mTOR	Mechanistic target of rapamycin
mTORC1	Mechanistic target of rapamycin complex 1
NF-κB	Nuclear factor kappa B
NFIB	Nuclear factor I B
NSCLC	Non-small cell lung cancer
PACMA	Propynoic acid carbamoyl methyl amide
PD-1	Programmed cell death protein 1
PD-L1	Programmed death-ligand 1
PDI	Protein disulfide isomerase
PDIA1	Protein disulfide isomerase A1
PDIA3	Protein disulfide isomerase A3
PDIA4	Protein disulfide isomerase A4
PDIA6	Protein disulfide isomerase A6
PERK	Protein kinase R–like ER kinase
PI3K	Phosphoinositide 3-kinase
PPARδ	Peroxisome proliferator-activated receptor delta
PRDX4	Peroxiredoxin 4
ROS	Reactive oxygen species
S1PR1	Sphingosine-1-phosphate receptor 1
SLC7A11	Solute carrier family 7 member 11
SPR	Surface plasmon resonance
STAT3	Signal transducer and activator of transcription 3
TNBC	Triple-negative breast cancer
UPR	Unfolded protein response
VEGF-A	Vascular endothelial growth factor A
VEGFR2	Vascular endothelial growth factor receptor 2
